# GATA3 and Aging Raise the Susceptibility of Metastasis to High-Grade Serous Ovarian Carcinoma

**DOI:** 10.34172/apb.43915

**Published:** 2025-02-22

**Authors:** Mohnad Abdalla, Amr Ahmed El-Arabey, Zhongtao Gai

**Affiliations:** ^1^Pediatric Department, Research Institute of Pediatrics Children’s Hospital, Shandong University (Jinan Children’s Hospital), Jinan, China.; ^2^Center of Bee Research and its Products, King Khalid University, P.O. Box 9004, Abha 61413, Saudi Arabia.; ^3^Department of Health Specialties, Basic Sciences and their Applications, Applied College, King Khalid University, P.O. Box 9004, Abha 61413, Saudi Arabia.; ^4^Department of Pharmacology and Toxicology, Faculty of Pharmacy, Al-Azhar University, Cairo, 11751, Egypt.

## To Editor,

**Figure 1:**
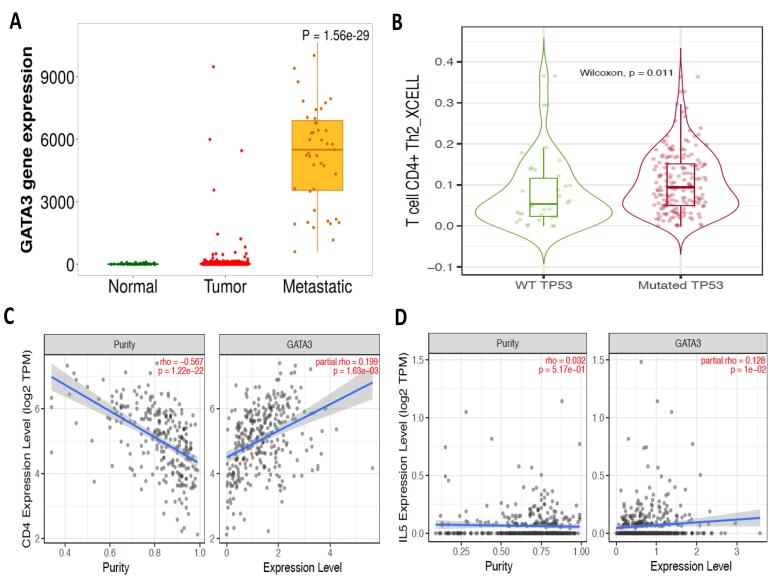
A bioinformatic analysis of HGSOC utilizing gene chip-based data and TNMplot.com found that GATA3 is much more frequent in metastatic areas than in normal or HGSOC (A). Bioinformatic analyses of the cancer genomic atlas (TCGA) database using TIMER2.0: revealed that mutant-TP53 HGSOC patients had substantially greater levels of T cell CD4 + Th2 compared to wild-type TP53 (p = 0.011) (B), GATA3 correlates positively with CD4 + (C) and IL-5 (D) in HGSOC patients

 Ovarian cancer (OC), particularly high-grade serous ovarian cancer (HGSOC), is a major health problem worldwide. It is commonly referred to as the “silent killer” since it can progress without causing any symptoms until it reaches an advanced stage. When it’s identified, it’s typically more difficult to treat properly. In the United States, HGSOC is the most common cause of gynecologic cancer mortality. Several reasons contribute to this, including a lack of efficient early detection screening technologies and the disease’s aggressiveness. Globally, OC is the sixth most often diagnosed cancer among women. Its prevalence highlights the significance of ongoing research into new diagnosis tools, treatment alternatives, and preventive actions to improve outcomes for people suffering from this condition.^1^ GATA binding protein-3 (GATA-3) is a versatile transcription factor required for the development and function of several tissues and cell types throughout the body. Its many functions underscore its importance in regulating several aspects of cellular differentiation and tissue specificity.^2^ GATA3-positive macrophages with an M2 phenotype are linked to fibrotic remodeling in the aged heart. Targeting particular subgroups of inflammatory cells, such as GATA3-positive macrophages, rather than overall inflammation, maybe a more effective method for treating fibrotic disorders linked with aging.^3^ The expression of the transcription factor gene GATA-3 in lymphocytes rises with age. Women have much greater expression of GATA-3 than males.^4^ Tumor Protein p53 (TP53) inhibits oncogenesis by controlling the expression of genes involved in apoptosis, metabolism, DNA repair, and cell cycle arrest. Increasing data shows that TP53 works as a tumor suppressor during inflammatory microenvironmental reactions. TP53 mutations can shield cancer cells from contact with the tumor microenvironment (TME) and the immune system, increasing tumor growth. TP53 mutations can also cause inflammation in response to inflammatory cytokines/chemokines and infections.^1^ A study has found a negative relationship between GATA3, the master regulator of macrophage polarization, and TP53 in patients with HGSOC.^1^ The interaction of tumor associated macrophages (TAMs) and mutant TP53 in OC boosts GATA3 expression, implying that mutant TP53 orchestrates macrophage infiltration in OC patients. Mutant TP53 and its co-regulators might be future therapeutic targets for OC elimination.^1^ TP53 mutations account for a major amount of the rise in cancer incidence rates linked with aging. Emerging data suggests that TP53 mutations have a causal role in the age-related rise in cancer incidence.^5^ Normal aging leads to an increase in CD4 + CD294 + Th2 cells. Aging has a deleterious impact on CD3 + T cells, cytotoxic T cells, and T helper cells.^6^ With aging, mononuclear cells produce more interleukin-5 (IL-5). Reduced production of Th-1 type cytokines, along with normal or enhanced production of Th-2 type cytokines, may contribute to reported immune response patterns in the elderly, such as a normal or increased humoral response and low cell-mediated immunity.^7^ These researches give information on the intricate interplay between aging, immune system changes, inflammation, and disease vulnerability, potentially informing future treatment methods for age-related disorders. In mouse models, aging raises the risk of OC metastasis. In this regard, age-related alterations in tumor-infiltrating lymphocytes (TILs) and B cell-related pathways in adipose tissue may lead to an increase in metastatic tumor burden in elderly hosts.^8^ Here, we would like to highlight the potential mechanism of GATA3 to promote the susceptibility of metastases in HGSOC related to aging. To demonstrate that we used TNMplot.com to compare normal, malignant, and metastatic research, we performed a thorough study on GATA3 in ovarian tissue utilizing gene chip-based data.^9^ Our investigation revealed that GATA3 is substantially more prevalent in metastatic sites than in normal and HGSOC (Figure 1A). Next, we utilized TIMER2.0,^10^ which provides a complete platform to investigate and display how TP53 mutation influences immune cell infiltration in HGSOC and assess their clinical impact by using The Cancer Genomic Atlas (TCGA) database. Our results showed that mutant-TP53 HGSOC patients exhibit increased levels of T cell CD4 + Th2 compared to wild-type TP53 (Figure 1B). Furthermore, GATA3 is positively correlated with CD4 + and IL-5 in HGSOC patients (Figure 1 C and D). As a result, GATA3 and aging may enhance the HGSOC metastasis via tumor-infiltrating CD4 + Th2 cells.

## Conclusion

 GATA3 expression is higher in metastatic regions than in normal and primary HGSOC tissues, indicating that it plays an important role in metastasis, which may be mediated by tumor-infiltrating CD4+ Th2 cells. Mutant TP53 in HGSOC corresponds with enhanced CD4+ Th2 cell infiltration and interacts with TAMs to upregulate GATA3, resulting in a tumor-promoting milieu. Age-related immunological changes, such as Th2 dominance, IL-5 overproduction, and decreased cell-mediated immunity, may worsen HGSOC metastasis by creating a favourable environment for tumour growth. Targeting mutant TP53, its co-regulators, or GATA3-driven pathways such as Th2 polarisation may provide innovative options for disrupting macrophage infiltration, immune evasion, and metastasis in HGSOC, particularly in aging populations. Besides, more research into the GATA3-TP53 axis and aging-related immunological alterations is required to develop early detection tools and precision medicines for better outcomes in HGSOC.

## Competing Interests

 The authors declare that they have no known competing financial interests or personal relationships that could have appeared to influence the work reported in this paper.

## Ethical Approval

 Not applicable.
